# The Indirect Relationship Between Spiritual Experiences and Subjective Wellbeing Through Hope? A Sample of Chilean Students

**DOI:** 10.1007/s10943-021-01459-4

**Published:** 2021-11-19

**Authors:** Marcin Wnuk

**Affiliations:** Department of Work and Organizational Psychology, Adam Mickiewicz University, Szamarzewskiego 89AB, 60-568 Poznań, Poland

**Keywords:** Spirituality, Religiousness, Religious practices, Hope, Subjective wellbeing

## Abstract

Spirituality and religiousness are important factors for adolescents wellbeing. Little is known about the mechanisms underlying the positive relationship between spirituality as well as religiousness and subjective wellbeing. This study aimed to verify, whether, in a sample of Chilean students, religiousness is indirectly related to hope through spiritual experiences, and whether spiritual experiences are indirectly related to subjective wellbeing via hope. The sample consisted of 177 Chilean students and the following measures were applied: the Daily Spiritual Experiences Scale, the Herth Hope Index, the Satisfaction With Life Scale, the Positive and Negative Affect Schedule, and one item measuring the frequencies of prayer and Mass attendance. According to obtained results religiousness was indirectly, positively related to hope through spiritual experiences. In turn spiritual experiences were indirectly, positively related to subjective wellbeing through hope. Conducted research confirmed the beneficial role of religious practices, spiritual experiences, and hope for Chilean students' subjective wellbeing and the presence of mechanisms underlying the relationships between religiousness as well as spirituality and subjective wellbeing.

## Introduction

In recent years, a growing body of research has focused on the religious and spiritual factors that influence health and wellbeing **(**Cohen & Koenig, 2003; Hill & Pargament, 2008; Jordan et al., 2014; Park, 2007; Seybold & Hill, 2001; Siegel et al., 2001) in an attempt to explain both the positive and negative impacts of religiousness and spirituality on physical, social, and psychological functioning (Chatters, 2000; Weber & Pargament, 2014). Researchers have attempted to identify and describe the mechanisms underlying the relationship between spirituality/religiousness and wellbeing by examining genetic, biological, social, and psychological factors (Baetz & Toews, 2009). Mostly in the role of potential mediators of spirituality/religiousness and wellbeing relationships have verified psychosocial variables, such as purpose and meaning in life (Aghababaei & Błachnio, 2014; Chamberlain & Zika, 1988; Park et al., 2008; Steger & Frazier, 2005; Vilchensky & Kravetz, 2005; Wnuk & Marcinkowski, 2014; Yoon et al., 2015), social support (Holt et al., 2005; Nooney & Woodrum, 2002; Prado et al., 2004), coping (Canada et al., 2006; Holt et al., 2005; Prado et al., 2004), religious coping (Fabricatore et al., 2004; Nooney & Woodrum, 2002; Schaefer & Gorsuch, 1991; Watlington & Murphy, 2006), optimism (Aglozo et al., 2021; Cheadle & Schetter, 2018; Kvande et al., 2015; Warren et al., 2015), and forgiveness (Lawler-Row, 2010).

Relatively, less is known about the role of hope in the relationship between religious or spiritual involvement, and subjective wellbeing. Various studies conducted upon students in different countries have indicated that spirituality and religiousness can indirectly improve wellbeing through hope (Chang et al., 2016; Marques et al., 2013; Nell & Rothmann, 2018; Wnuk & Marcinkowski, 2014). The aim of the study was to verify, whether in a sample of Chilean students religiousness is indirectly related to hope through spiritual experiences, and, whether spiritual experiences is indirectly related to subjective wellbeing through hope.

Chile is a historically Catholic country. At present, the majority of Chileans self-identify as Roman Catholic. Furthermore, 70% of Chileans self-identify as being “highly religious,” meaning that religion plays a significant role in their lives (Vázquez & Páez, 2011).

Relationships between religiousness and spirituality as well as subjective wellbeing depend not only on the measures used to verify them (Poloma & Pendleton, 1990), but also on socioeconomic and cultural factors (Diener et al., 2011; Lun & Bond, 2013). For example, in religious nations, religious individuals have higher rates of subjective wellbeing, although, non-religious people have higher rates of subjective wellbeing in non-religious nations (Diener et al., 2011; Graham & Crown, 2014; Stavrova et al., 2013). Also, in national cultures where socialization of religious faith is more common, spiritual practices are positively related to subjective wellbeing, whereas, in cultures where religious socialization is less prevalent, the relationship between spiritual practices and subjective wellbeing is reversed (Lun & Bond, 2013). According to research conducted on the national level among individuals from 154 nations by Diener et al. (2011), the relationship between religiousness and the measures of subjective wellbeing are mediated by the resources provided by religion including social support and life purpose/meaning. Another function of religion is to deliver hope, which can be related to the indirect impact of religiousness on wellbeing.

Numerous studies have confirmed that spirituality and religiousness are important sources of hope (Conco, 1995; Coyle, 2002; Hong et al., 2015; Pargament, 2013). Considering that Chile is a very religious nation and the level of support for religious socialization is relatively high (Lun & Bond, 2013), it can be expected that, among Chilean students, religious practices are positively correlated with spiritual experiences, which in turn are indirectly related to subjective wellbeing through hope.

Practicing-religious people differ from non-religious people and non-practicing religious people with regard to hope as a strength of character. Compared with members of two other groups, practicing-religious individuals scored higher in hope (Berthold & Ruch, 2014). According to Ivtzan et al. (2013), people can have a high level of religious involvement and spirituality (1), a low level of religious involvement with a high level of spirituality (2), a high level of religious involvement with a low level of spirituality (3), or a low level of religious involvement and spirituality (4). Aside from a few exceptions, participants from groups one and two had higher levels of psychological wellbeing, measured self-actualization, meaning in life, and personal growth initiative (Ivtzan et al., 2013). In the line with these results, Chilean students with declared religious affiliations can be expected to have spiritual experiences because of their religious practices but non-affiliated, agnostic, and atheist students may also achieve spiritual experiences through the use of secular practices such as meditation, contemplation, and mindfulness (Cobb et al., 2015; Labelle et al., 2015; Wachholtz & Pargament, 2005). Additionally, prayer can be used as a coping resource among non-believers in difficult life situations and times of crisis.

## Religiousness, and Spirituality Similarities and Differences; Their Role in Explaining Hope

Religiousness and spirituality is an area of research explored in many academic and scientific disciplines including sociology, psychology, ethics, medicine, nursing, anthropology, etc. Many researchers have explored the functions of religion and spirituality in the daily lives of individuals, along with the impact of religion and spirituality on health and wellbeing (Sawatzky et al., 2005; Koenig, 2001; Park, 2007; Seybold & Hill, 2001).

Both religiousness and spirituality are multidimensional and multifaceted constructs (Hill et al., 2000; Fetzer Institute/National Institute on Aging, 1999; Demmrich & Huber, 2019; Saucier & Skrzypińska, 2006) and well-established discussions comparing their similarities and differences are lacking in the literature reviewed in this study (Gall et al., 2011; George et al., 2000; Hodge & McGrew, 2005). Historically, spirituality has its own roots in religion and remains an element of religiousness (Wulff, 1991). Consequently, spirituality's increase in popularity has come at the expense of religiousness (Ribaudo & Takahashi, 2008). The best indicator of this process is the data that shows between 1965 and 2000, there was a significant increase in health-related articles dealing with spirituality and a corresponding decrease in health-related articles addressing religion (Weaver et al., 2006).

According to Pargament (1999), religion currently has a negative connotation and is associated with institutional, ritual-based, and ideological practices, in contrast to spirituality, which is defined as a non-static entity and a dynamic, positive process that focuses on individual and personal feelings, experiences, and thoughts.

In the literature examined in this study, there is no coherent or straightforward approach to analyzing the similarities and differences of both concepts although some general findings are available. First, regardless of the research samples, participants view religiousness and spirituality as separate but overlapping constructs more than the same or completely different phenomena (Baumsteiger & Chenneville, 2015; Hodge & McGrew, 2005; Hyman & Handal, 2006). For example, in a study by Zinnbauer et al. (1997), self-rated spirituality correlated positively with self-rated religiousness. In another study, 60% of research students confirmed that a relationship exists between spirituality and religion (Hodge & McGrew, 2005). While spirituality is a broader concept than religion, religiousness is a form of spirituality and spirituality includes religion (Baumsteiger & Chenneville, 2015; Hyman & Handal, 2006). In the surveyed research, participants more often identified with a definition of spirituality as internal, individual, and subjective (Baumsteiger & Chenneville, 2015; Hyman & Handal, 2006) as opposed to religion as external, collective, and objective (Baumsteiger & Chenneville, 2015; Hyman & Handal, 2006).

Additionally, compared to spirituality, religion is more frequently indicated as a theistic practice (Schlehofer et al., 2008), one connected with organized spiritual practices like rituals, worship, etc. (Baumsteiger & Chenneville, 2015; Hodge & McGrew, 2005; Hyman & Handal, 2006) that possesses negative connotations (Baumsteiger & Chenneville, 2015; Schlehofer et al., 2008).

Religion, whether in traditional or non-traditional forms, provides a supportive context for spiritual growth (Hill & Pargament, 2003; Davis et al., 2015). Recent studies have confirmed that most aspects of religiousness are related to spirituality (Heintz & Baruss, 2001). In a study by Zinnbauer et al. (1997), participants' self-rated spirituality was positively related to respondents' frequency of prayer and church attendance.

Religious practices are positively correlated with spiritual experiences. For example, in a study conducted by Park et al. (2012) on a representative sample of American adults, a four-class model was identified based on spiritual and religious involvement: highly religious, moderately religious, somewhat religious, and minimally religious or non-religious. Compared with members of other groups, highly religious individuals scored higher in prayer, attending religious services, positive religious coping, and daily spiritual experiences. Johnson et al. (2008) conducted a factor analysis using different measures of spirituality and religiousness and observed that spiritual experiences strongly correlate with religious practices such as prayer and church attendance. American adults with organized and private religious practices had positive relations with spiritual experiences (Yoon et al., 2015). In a sample of Salvadoran youth, religiousness (consisting of inter alia religious event participation) positively predicted spirituality (King et al., 2020), which, in turn, improved hope. Religious practices indirectly influenced hope through spiritual experiences among Polish, codependent, female members of Al-Anon (Wnuk, 2015) as well as in a sample of members of Alcoholics Anonymous in Poland (Wnuk, 2021).

### Hypothesis 1

In a sample of Chilean students, religious practices are indirectly related to hope through spiritual experiences.

### Hope

In psychology, hope is considered to be a strength of character that is part of transcendence virtue (Seligman, Peterson, & Park, 2004a; Seligman, Peterson, & Park, 2004b), trait or state (Snyder, 2002), as well as emotion (Lazarus, 1999). Despite their differing approaches, researchers underline the multidimensional character of hope (Scioli et al., 2011). The subject of hope is of importance and value to the individual. Hope is reflected in one's thoughts, feelings, behaviors, and relations with other people; it consists of an element of prediction, making it future-oriented but rooted in the present and connected to the past (Stephenson, 1991).

According to Kozielecki (2006), hope consists of cognitive, emotional, affiliative, temporal, and causative factors. The cognitive dimension of hope relates to convictions about goal-reaching, obtaining a significant result, and/or value realizations. These convictions are “saturated with emotions and feelings that influence the perception of the attractiveness of the subject of hope, thus constituting its emotional aspect. The affiliative factor of hope is concerned with people who, through their behavior, help to create or maintain hope for reaching their desired goal. Temporal aspect of hope is defined as a “concentrating on future but based on experiences from the past and present” (Stephenson, 1991).

The causative dimension of hope has a motivational role in maintaining activity and behaviors directed at reaching the desired goal. Kozielecki (2006) divides hope into four types based on two main dimensions. On one end of the continuum, he places “particular hope,” with “generalized hope” on the opposite end. Hope may have an active or passive character. “Generalized hope” does not concern any concrete goal. “Particular hope” is focused on a specific result. Hope with a passive character activates assumptions that no engagement is happening in any activity, adhering to an assumption that even though there is no activity toward reaching the goal, the goal will be reached. Hope leads to actions that are focused on achieving a goal. The main strategies that help maintain hope are the presence of significant relationships with other people and the ability to experience ease. Possessing attributes like determination, courage and serenity, clear goals, spiritual faith, the ability to summon positive memories, and having respect for and acceptation of others' individuality all facilitate the hope-maintaining and developing process (Herth, 1990). This study adopts the definition of hope as a trait of character. According to this definition, “hope is a multidimensional, dynamic life force that can be characterized as a certainty that reaching “good” is possible and that a personally valuable and significant goal is achievable” (Dufault & Martochio, 1985).

## The Indirect Relationship Between Religiousness/Spirituality and Subjective Wellbeing Through Hope

The positive impact that religiousness/spirituality has on shaping hope has been confirmed in studies of oncology patients (Bowes et al., 2002; Fehring et al., 1997; Herth, 1989), family members looking after their terminally-ill relatives (Herth, 1993), terminally-ill patients (Buckley & Herth, 2004), codependent individuals participating in self-help groups (Wnuk, 2015), as well as students (Wnuk & Marcinkowski, 2014). Many more studies have confirmed the positive relationship between hope and subjective wellbeing. Subjective wellbeing refers to one's satisfaction with life and the extent to which one experiences positive feelings and/or general happiness (Diener, 2009). It is characterized by low levels of neuroticism or negative affect and concomitant high levels of positive affect (Diener, 2009). In a study by Proyer et al. (2011), among all strengths of character, hope had the strongest correlation to present and future life satisfaction. The same result was achieved in three internet samples of adults (Park et al., 2004a, 2004b). Similarly, Peterson et al. (2007) found that among both American and Swiss adults, the strength most highly linked to life satisfaction was hope. A longitudinal study by Ciarrochi et al. (2015) confirmed that hope is an antecedent to the positive as well as negative affect of adolescents.

Recent studies conducted among adolescents and students from different countries have emphasized that religious and spiritual variables are indirectly related to different wellbeing indicators through hope.

In a longitudinal study conducted on Portuguese adolescents, Marques et al. (2013) confirmed the mediating role of hope between religious practices and life satisfaction. According to a recent study of college students from USA, spirituality and religiosity indirectly influenced depressive symptoms through hope (Chang et al., 2016). The same effect was noticed in a study of students in Poland (Wnuk & Marcinkowski, 2014). In this research, more frequent spiritual experiences were correlated with a higher level of hope, which in turn was related to increased intensity of positive affect and life satisfaction.

There is no existing research about the spiritual/religious sphere of life and hope as it relates to Chilean students and its potential influence on their wellbeing. Recent studies conducted on a sample of Chilean adults have confirmed the positive role of spirituality and religiousness in wellbeing (Gallardo-Peralta, 2017; Fernández, Lorca & Valenzuel, 2020). This study has a purpose to fill this gap examining the mechanisms undelaying the relationship between religiousness as well as spirituality and subjective wellbeing.

### Hypothesis 2

In a sample of Chilean students, spiritual experiences are indirectly related to subjective wellbeing through hope.

## Method

### Participants

The research sample within this study consisted of university students from Chile. Questionnaires in Spanish language were given to students after classes and, upon completion, were collected by an overseas student with Polish grant funding. Among 180 distributed questionnaires, two students did not consent to participate in research and one student did not completely fill the questionnaires. Finally in research remainded 177 students. Due to the potential lack of harmful effect of participation in research agreement of ethical committee was not required. Descriptive statistics of socio-demographics variables were presented in Table 1. All participants had a secondary education and used Spanish language. From the religious affiliation sample was heterogeneous.

**Table 1:** Demographics variables in Chilean students sample (*n* = 177). (Source: author’s research)

	Classification	Percentage or Mean
Gender	Men	62%
	Women	38%
Age		21.35 years
Education	Secondary	100%
Languages	Spanish	100%
Denomination	Roman Catholic church	55,37%
	Evangelicalism	10.96%
	Seventh-day Adventists	8.3%
	Jehovah's witness	2.2%
	Mormons	1.1%
	Tribal religions	0.6%
	Atheists	6.7%
	Agnostics,	7.57%
	Non-affiliated	7.2%

### Measure

The Daily Spiritual Experiences Scale (DSES) consists of 16 questions, each with six points ranging from 1 (*never or almost never*) to 6 (*many times daily*). The Spanish language version of this tool was used (Mayoral et al., 2013). The more points scored, the greater the respondent's level of spirituality. Depending on population, the scale’s reliability ranges from *α* = 0.86 to 0.95 (Underwood, 2011). The short version of this measure was used, which consists of six items.

Two measurement scales concerning religious practices were used. The first measurement was based on a five-point scale and measured how frequently participants attended Mass. The options on this scale consisted of *never with the exception of baptisms, marriages, or funerals* (1), *a few times a year* (2), *1–2 times monthly* (3), *2–3 times monthly* (4), and *once per week or more* (5). The second scale measures respondents' frequency of prayer. Participants reported how often they prayed, with response options ranging from *never*, to *sometimes*, *once monthly*, *once weekly,* or *every day*.

The Positive and Negative Affects Schedule (PANAS) consists of ten statements related to positive emotional states and another ten concerning negative ones. Each question is graded from 1 = *a little or none* to 5 = *very frequently*. The more points scored, the greater a person’s negative as well as positive affect. Participants were asked to assess their emotional state based on how often they related to particular questions up to the weekend before the survey. The Spanish language version of this measure was used (Ortuño-Sierra et al., 2015). According to studies, the reliability scale varied from *α* = 0.86 to 0.89 for the positive affect and *α* = 0.84 to 0.85 for the negative affect (Crawford & Henry, 2004).

The Satisfaction with Life Scale (SWLS) is a universally-recognized tool used to measure one's mental wellbeing based on an operational concept of satisfaction with life, defined by a conscious assessment/judgment of one’s life compared to self-imposed standards (Diener et al., 1985). The Spanish language version of this measure was used (Vázquez et al., 2013). This measure consists of five statements that are graded according to a seven-point scale. According to this measure, the greater the points, the more satisfied the respondent is with life. This scale possesses satisfactory psychometric properties in this study. Its reliability is 0.83, as determined by the test–retest method after a two-week repeated study, which rose to 0.84 after a month but then ranged between 0.64 and 0.82 after two months (Pavot & Diener, 1993). The more points scored, the greater the respondent's life satisfaction. The method’s unequivocal nature has been confirmed by various studies (Diener et al., 1985; Pavot et al., 1991; Shevlin & Bunting, 1994).

The Hope Herth Index (HHI) is a scale used for the measurement of hope based on a particular definition of hope. The Spanish language version of this measure was used (Arnau, et al., 2010). Participants answered 12 questions expressed on the four-step Likert scale (1–4) ranging from 1 (*I strongly disagree*) to 4 (*I strongly agree*) (Herth, 1992). The reliability of this scale has satisfactory psychometrical features. In reference to patients' scores, *α* = 0.97 (Herth, 1992) were evaluated with the test–retest method score of 0.91 (Herth, 1992).

## Statistical Analysis

**Figure 1:**
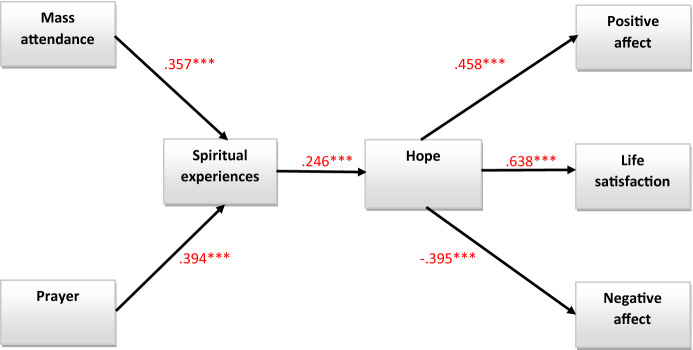
Model of research. *Note*. The standardized regression coefficients are presented. *p < .05, **p < .01, ***p < .001. For the sake of legibility, the correlations between the residuals were omitted in Fig. 1. ( Source: author’s research)

All statistical analyses were conducted using IBM SPSS Statistics software (Version 27.0). To verify potential multicollinearity problem values of Variance Inflation Factors (VIF) for research variables were computed. Structural equation modeling was used to test the hypothesis. A tested model reflecting the research hypotheses is shown in Fig. 1. The following model of fit indices was used: normed fit index (NFI), goodness of fit index (GFI), comparative fit index (CFI), and root mean square error of approximation (RMSEA). Critical values for good model fit have been recommended for the CFI to be acceptable above the 0 90 level and RMSEA should be 0 05 or less and should not exceed 0 08 (Wang & Wang, 2012). The level of NFI should exceed 0.90, as should the levels of GFI (0.90) and CFI (0.93) (Steiger, 1990). Also, based on the chi-square statistic, the values of CMIN/*df* statistics should be lower than the required standard, 2 or 3 (Byrne, 1994).

## Results

The descriptive statistics and reliability of the measurements used are presented in Table 2. The values of the r-Pearson correlation coefficients are presented in Table 3. There were no values of VIF exceeding 5 (see Table 2), thus, the multicollinearity problem was not likely to exist (Menard, 2001). The values of RMSEA = 0.000 (90% CI [0.000, 0.0039]), NFI = 0.982, GFI = 0.989, AGFI = 0.975, CFI = 1, chi^2^ = 6.73, *df* = 12, *p* = 0.895 (CMIN/*df* = 0.561) indicated that the fit between the measurement model and the data were acceptable. Due to the relatively small sample size, the Bollen–Stine bootstrapping method was used to increase the likelihood of veracity of the obtained results and verified direct and indirect effects. The Bollen–Stine bootstrapping method (*p* = 0.881) performed on 5,000 samples confirmed the good fit of the tested model.

**Table 2:** Descriptive statistics and reliability of scales in Chilean students sample (*n* = 177). ( Source: author’s research)

Measures	Mean	SD	Skewness	Kurtosis	Minimum	Maximum	VIF	Reliability
Prayer	2.84	1.47	0.298	− 1.413	1	5	1.85	
Mass attendance	2.81	1.90	0.980	− 0.192	1	5	1.80	
DSES	18.22	6.74	0.074	− 0.907	6	35	1.06	0.85
PANAS								
Positive affect	16.47	3.51	0.169	0.242	6	25		0.72
Negative affect	14.06	4.22	0.685	0.260	5	25		0.82
SWLS	27.04	5.41	− 1.188	1.147	6	35		0.83
HHI	36.76	4.62	− 0.240	1.122	24	55	1.88	0.77

DSES—Daily Spiritual Experiences Scale; PANAS—Positive and Negative Affect Schedule; SWLS—Satisfaction with Life Scale; HHI—Herth Hope Index, VIF—Variance inflation factor

**Table 3:** Correlation matrix in Chilean students sample *(n* = *177).* ( Source: author’s research)

	1	2	3	4	5	6
1. Life satisfaction						
2. Positive affect	0.47**					
3. Negative affect	− 0.38**	− 0.15				
4. Spiritual experiences	0.24**	0.14	− 0.10			
5. Hope	0.64**	0.46**	0.39**	0.25**		
6. Prayer	0.13	0.01	− 0.05	0.61**	0.16*	
7. Mass attendance	0.16*	0.01	− 0.04	0.59**	0.17*	0.60**

*p ≤ 0,05

**p ≤ 0,01

The standardized direct effects of prayer on spiritual experiences and Mass attendance on spiritual experiences were statistically significant, respectively, (CI 95% [0.257; 0.519], *β* = 0.394, *p* = 0.000) and (CI 95% [0.219; 0.485], *β* = 0.357, *p* = 0.000). The standardized direct effects of spiritual experiences on hope was statistically significant (CI 95% [0.106; 0.385], *β* = 0.246, *p* = 0.001) and hope in turn were statistically significantly related to life satisfaction (CI 95% [0.539; 0.716], *β* = 0.638, *p* = 0.000), positive affect (CI 95% [0.322; 0.569], *β* = 0.458, *p* = 0.001), and negative affect (CI 95% [ − 0.515;  − 0.263], *β* = − 0.395, *p* = 0.000).

The indirect effects of prayer through spiritual experiences on hope was statistically significant 0.10, *p* = 0.001 (CI 95% [0.040; 0.169],), the same as indirect effect of prayer on life satisfaction 0.06, *p* = 0.001 (CI 95% [0.26; 0.113]), indirect effect of prayer on positive affect 0.04, *p* = 0.001 (CI 95% [0.181; 0.84]) as well as indirect effect of prayer on negative affect -0.04, *p* = 0.001 (CI 95% [ − 0.76; − 0.16]).

Also noticed statistically significant indirect effects of Mass attendance on spiritual experiences through hope 0.09, *p* = 0.001 (CI 95% [0.037; 0.163]), the same as indirect effect of Mass attendance on satisfaction with life 0.06, *p* = 0.001 (CI 95% [0.023; 0.106]), indirect effect of Mass attendance on positive affect 0.04, *p* = 0.001 (CI 95% [0.16; 0.78]), as well as indirect effect of Mass attendance on negative affect − 0.3, *p* = 0.001 (CI 95% [ − 0.065; − 0.017]).

Additionally spiritual experiences were statistically significantly, indirectly through hope related to life satisfaction 0.16, *p* = 0.001 (CI 95% [0.066; 0.256]), as well as positive and negative affect, respectively, 0.11, *p* = 0.001 (CI 95% [0.048; 0.194]) and − 0.10, *p* = 0.001 (CI 95% [ − 0.041; − 0.173]).

## Discussion

This study aimed to examine two indirect mechanisms in the relationship between religious practices and hope through spiritual experiences and in the relationship between spiritual experiences and subjective wellbeing through hope. According to obtained results, the relationship between spiritual experiences and subjective wellbeing of the Chilean students is indirect, and the variable being underlying this relation is hope. This means that, for Chilean students, spiritual experiences are a positive predictor of their level of hope, which in turn is related to increased life satisfaction and positive affect and decreased negative affect. These results are largely consistent with the recent studies conducted among youth and their family members from Republic of South Africa (Nell & Rothmann, 2018), as well as students from Poland (Wnuk & Marcinkowski, 2014), students from USA (Chang et al., 2016), adults from the USA (Chang et al., 2013), members of Alcoholics Anonymous from Poland (Wnuk, 2021) and oncology patients (Zarzycka et al., 2019). These findings suggest that the mechanism of spirituality's indirect relationship with subjective wellbeing through hope can be universal, independent of the conceptualization of these constructs, the measures used, cultural contexts, and research sample populations.

In explanation of the observed mechanism is worth focusing on youth individuals and students as a comparable sample. In comparison with the study by Wnuk and Marcinkowski (2014), another measure of life satisfaction was used, short instead of normal versions of the Daily Spiritual Experiences Scale was applied, sample was heterogeneous regarding religious denominations and it was preserved balance between men and women. Among the students surveyed from Poland, all were Roman Catholic. Regardless of the noticed differences in both samples, spiritual experiences were indirectly related to subjective wellbeing via hope. Although among Polish students, spiritual experiences were not indirectly related to negative affect, this discrepancy could be explained using another older and not as reliable verification method of indirect effect (Baron & Kenny, 1986).

It is worth noting that there was no statistically significant correlation between spiritual experiences and negative affect in Chilean students (see Table 3), and the use of structural equation modeling revealed that this relationship is indirect. In a Wnuk and Marcinkowski study (2014), the strength of the r-Pearson correlation between spiritual experiences and negative affect was not statistically significant but it was twice as large as the correlation for Chilean students, meaning that the use of SEM instead of multiple regression analysis could confirm that this association is indirect.

Also, a sample of students from Republic of South Africa that used another measure of hope and religiousness confirmed that religious involvement leads to hope, which in turn is positively correlated with life satisfaction as well as positive affect and negatively related to negative affect (Nell & Rothmann, 2018).

In every analyzed group of research participants (Chang et al., 2016; Nell & Rothmann, 2018; Wnuk & Marcinkowski, 2014), religious socialization was rather prevalent and on a comparable level (Lun & Bond, 2013) to the level of religiousness (Diener et al., 2011; Graham & Crown, 2014; Stavrova et al., 2013) as a potential source of differences in results. This means that this cultural factor was not significant from the perspective of potential differences in research results.

The hypothesis that religious practices are indirectly related to hope via spiritual experiences was fully confirmed. This means that in the sample of Chilean students, prayer and Mass attendance are correlated with spiritual experiences, which in turn are related to hope. The results of this study are consistent with recent research (Wnuk, 2015; King et al., 2020) that confirmed an indirect relationship between different facets of religiousness and hope via spiritual experiences. In Wnuk's study (2015), in a sample of codependent individuals participating in Al-Anon, apart from private and public indicators of religiosity such as prayer and Mass attendance, other religious facets such as strength of faith and religious coping were indirectly related to hope through spiritual experiences. It is important to note that in comparison to the Wnuk study (2015), the same measures of hope, religiousness, and spiritual experiences were used in this study. There was only one difference regarding the use of the Daily Spiritual Experiences Scale's short form.

Consistent with data from another research, religious practices are probably significant ways lead to spiritual growth (Johnson et al., 2008; Yoon et al., 2015; Zinnbauer et al., 1997). Among Chilean students, prayer and Mass attendance were positively associated with spiritual experiences. The obtained results show that religiousness, measured by religious practices, and spirituality, measured by spiritual experiences, are rather separate but overlapping constructs (Baumsteiger & Chenneville, 2015; Hodge & McGrew, 2005; Hyman & Handal, 2006). The strength of relationships between prayer and spiritual experiences as well as Mass attendance and spiritual experiences were moderate. Spiritual experiences were directly related to hope but religious practices were only indirectly related to hope via spiritual experiences.

Additionally, spirituality, operationalized as a spiritual experiences, seems to be a wider concept than religiousness, understood as a religious practices. Chilean students may be using other secular practices that can lead to spiritual experiences such as meditation, contemplation, and mindfulness (Cobb et al., 2015; Labelle et al., 2015; Wachholtz & Pargament, 2005). Considering that many Chilean students declared themselves to be atheists and agnostics does not mean that religious-affiliated students cannot use secular practices and exercises to spiritual growth. Further research examining not only religious but additionally secular practices could verify this assumption.

It is important to emphasize that the Daily Spiritual Experiences Scale used to examine spiritual experiences is the measure that reflects the approach to religiousness and spirituality as overlapping constructs, independent from religious denominations. Daily Spiritual Experiences Scale contains items that are more specifically theistic in nature but also remains appropriate for those who are not comfortable with theistic language (Underwood, 2011).

For Chilean students, prayer and Mass attendance are connected with feeling God’s presence, finding strength in religion or spirituality, feeling deep inner peace or harmony, feeling God’s love through others, feeling spiritually touched by the beauty of creation, and a desire to be closer to God or in union with the divine. These spiritual experiences are probably the source of their hope and are reflected through their positive outlook toward life, their capability to recall happy/joyful times, feelings that life has value and worth, maintaining a sense of direction, lacking fear of the future, not feeling alone, having a deep inner strength, and believing that each day has potential. In turn, hope is for them a condition for living a satisfied and happy life. For Chilean adolescents, hope as a result of spiritual experiences is an important mental and emotional force in everyday life that aids them in their struggles with emerging adulthood goals, conflicts, problems, and difficulties connected with this period of life.

The conducted research yields some theoretical and practical implications. In a sample of Chilean students, religious practices were indirectly related to hope through spiritual experiences, and subjective wellbeing via the path of spiritual experiences and hope. Also, spiritual experiences indirect effect on subjective wellbeing through hope was recognized.

This predictive role of hope for Chilean students' subjective wellbeing indicates a need to implement hope-based interventions and programs for this population. It is a practical incentive for therapists and counselors to engage in preparing and implementing these kinds of intervention tools. Some studies have confirmed the efficiency of hope-based intervention programs for different facets of wellbeing, especially depression (Chan et al., 2019; Cheavens et al., 2006; Retnowati et al., 2015; Shekarabi-Ahari et al., 2012). The finding that religious practices probably indirectly influence subjective wellbeing can be of practical use to therapists and counselors working with religious students or within pastoral contexts. This means that religious-affiliated students can be encouraged by therapists and counselors to become involved in religious practices to improve their subjective wellbeing. Tailoring spiritual intervention focus on spiritual growth can lead to enhance students' wellbeing via hope. Recent research has indicated that religious and spiritual interventions in randomized, controlled clinical trials lead to positive outcomes such as decrease stress, alcoholism, and depression (Gonçalves et al., 2015).

## Limitations and Future Research

### This Study has Several Limitations

First, this study was based on students (i.e., young adults), which means that the generalizability of the findings is limited compared to the general student population of Chile. It is important to verify if similar or different findings can be revealed among diverse racial/ethnic groups (Gillum & Griffith, 2010) or in different cultural (Chang et al., 2016; Marques et al., 2013; Nell & Rothmann, 2018; Wnuk & Marcinkowski, 2014) and socio-economical (Diener et al., 2011; Lun & Bond, 2013) contexts. Recent studies have indicated that religion can play a positive role in wellbeing among populations that present high levels of religiousness (Diener et al., 2011; Graham & Crown, 2014; Stavrova et al., 2013) with relatively bad social conditions (Diener et al., 2011), where religious socialization is more prevalent and, unexpectedly, where social hostility toward religious groups is more intense (Lun & Bond, 2013). It is also important to verify if the present findings can be generalized across respondents from specific religious denominations (i.e., Catholics, Buddhists, Jews, and Muslims). In a study conducted on individuals affiliated with one of the four major religions (Buddhism, Christianity, Hinduism, and Islam), Diener, Tay, and Myers found that the relational patterns within the religiousness–wellbeing relationship are more similar than distinct (2011).

Because these findings must be interpreted as mainly applying to young adults, additional research is necessary to investigate the strength and nature of these relationships in samples among children, older adults, and geriatric populations. Considering that the SEM requires a large sample of participants, this research group was relatively small but the use of the bootstrapping method balanced this disproportion. This research was cross sectional, not longitudinal, which is why the described relationships cannot be analyzed from the cause-and-effect perspective. Another direction in the relationships between spiritual experiences, hope, and subjective wellbeing cannot be eliminated. It is possible that spiritual experiences is also positively correlated with subjective wellbeing, which in turn is positively connected with hope.

It would be interesting to verify the indirect impact of spirituality on wellbeing using hope as a bi-dimensional (Snyder, 2002) construct. An investigation of this nature could be especially important considering that the results of recent studies in this area remain inconsistent. For example, Nell and Rothmann (2018) found that both pathway and agency hope mediate the relationships between religiosity and life satisfaction, positive affect, and negative affect. The same pattern was revealed in a study by Chang et al. (2013) that used depressive symptoms as a dependent variable and spirituality as well as religiosity as independent variables. In another study conducted by Chang et al. (2016) on a different sample, only hope agency was found to be a mediator between ritualistic, theistic, and existential spirituality and depressive symptoms. This study was also limited by its use of a uni-dimensional measure of spirituality. Future research should focus on using another measures of spirituality, especially multidimensional measures, to examine if every facet of spirituality indirectly influences subjective wellbeing via hope.

In this study, only two indicators of religiousness were used (prayer and Mass attendance) as potential sources of spiritual experiences. It would be interesting to explore another religious practice like reading the Bible, or another religious indicators such as religious orientation, religious faith, or religious coping as antecedents of spirituality. Also examining additional non-religious, secular sources of potential spiritual experiences such as meditation, and contemplation (Davis et al., 2015) could provide interesting results.
